# Focusing on rare diseases in China: are we there yet?

**DOI:** 10.1186/s13023-015-0361-3

**Published:** 2015-11-02

**Authors:** Li Yang, Chang Su, Ashley M. Lee, Harrison X. Bai

**Affiliations:** Department of Neurology, The Second Xiangya Hospital, Central South University, No. 139 Middle Renmin Road, Changsha, Hunan 410011 PR China; Department of Bioengineering, University of Pennsylvania, 210S. 33rd street, Suite 240 Skirkanich Hall, Philadelphia, PA 19104 USA; Mallinckrodt Institude of Radiology, Washingto University in Saint Louis, 510 S Kingshighway Blvd, St. Louis, MO 63110 USA; Department of Radiology, Hospital of the University of Pennsylvania, 3400 Spruce Street, Philadelphia, PA 19104 USA

**Keywords:** Rare disease, China, Research papers, Clinical trials

## Abstract

The Chinese researchers have made significant progress in studying rare diseases in the recent years. From 2000 to 2014, 269 out of 1892 clinically relevant original research papers published on high impact journals by Chinese institutions, and 2678 out of 6040 clinical trials conducted by Chinese institutions and registered at ClinicalTrial.gov are focused on rare diseases. The number of research papers and of clinical trials has shown a steady trend of increase. Creating public databases for rare disease will escalate progress in rare disease and enable multicenter studies.

## Introduction

Rare diseases have been receiving increasing public attention in China [1]. Currently, approximately 15.6 million people in China are afflicted with rare diseases [2], a relatively large patient population that can contribute to and benefit from medical research. The Chinese government has taken steps in promoting rare disease awareness, facilitating medical research, and providing patient resources. Examples are the upcoming establishment of a Rare Diseases Prevention and Treatment Law and the foundation of the Chinese Organization for Rare Disorders [3]. In this letter, we would like to highlight the tremendous amount of progress that has been made by Chinese researchers in the recent years and additional steps that are currently underway to facilitate future research and collaboration.

## Methods and results

**Fig. 1:**
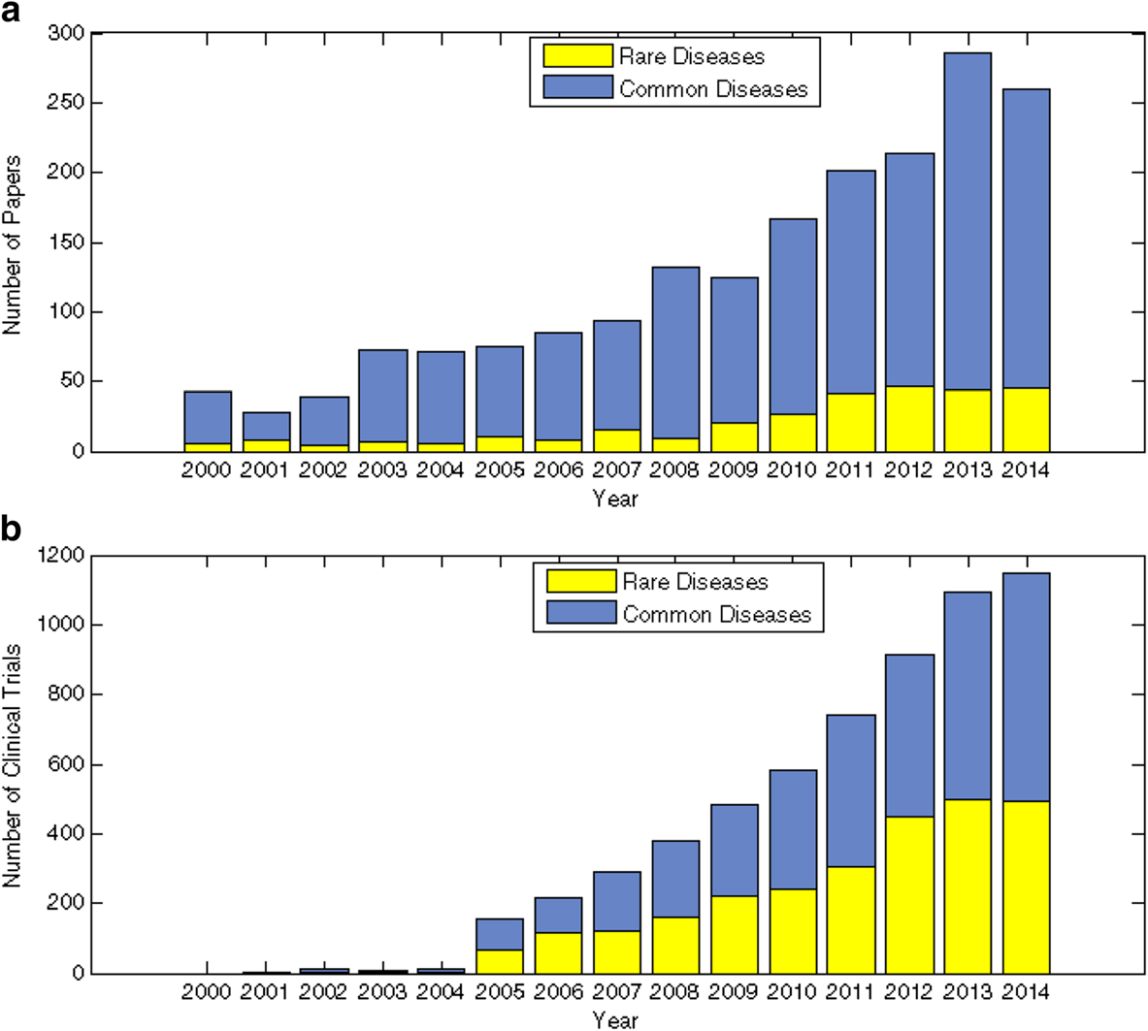
**a** The number of original research papers published by Chinese institutions in high impact journals. Letters, comments, and review articles were excluded. **b** The number of clinical trials registered by Chinese institutions in a given year at ClinicalTrials.gov. Trials are grouped by the year of registration

First, we searched pubmed.org for original research articles published by Chinese institutions in high impact clinical journals from 2000 to 2014. Articles were included if they (1) were published in a journal with clinical focus and an impact factor above 10, (2) listed China as its first affiliation in authors information, (3) studied human subjects or used human tissues. Per the European Commission on Public Health, rare diseases are defined as “life-threatening or chronically debilitating diseases which are of low prevalence”, or 1 in 2000 people [4]. Overall, 38 journals were included in our search. There were 1892 clinically relevant papers, 269 (20 %) of which studied rare diseases. There has been a steady trend of increase in recent years (Fig. 1a). Using the criteria above, out of the 21 studies published by Chinese institutions in the Orphanet Journal of Rare Diseases from 2012 to 2015, 11 studies used human subjects or tissues.

Second, we looked at clinical trials that were conducted by Chinese institutions and registered at ClinicalTrials.gov from 2000 to 2014. There was a total of 6040 clinical trials, 2678 (44 %) of which studied rare diseases. There has been an exponential increase of clinical trials since 2004 (Fig. 1b).

## Conclusion

Evidently, multiple centers in China have been studying rare diseases, resulting in a tremendous increase in the number of high-quality publications and clinical trials. However, most of the data are only available to local researchers. There is a need for open data access and a nationwide data-sharing platform. Examples of public database include The Cancer Genome Atlas in the United States and The European Genome-phenome Archive in Europe [5]. Creating a public database is a daunting task that requires collaborative efforts by researchers in the Chinese medical research community. However, its establishment will bring drastic change to the research environment in China, creating abundant opportunities for multi-center studies.
